# Effects of Upper-Limb Exoskeletons Designed for Use in the Working Environment—A Literature Review

**DOI:** 10.3389/frobt.2022.858893

**Published:** 2022-04-29

**Authors:** Tobias Moeller, Janina Krell-Roesch, Alexander Woll, Thorsten Stein

**Affiliations:** Karlsruhe Institute of Technology, Institute of Sports and Sports Science, Karlsruhe, Germany

**Keywords:** exoskeleton, wearable robotics, wearable device, overhead work, evaluation, occupational, industry

## Abstract

**Introduction:** Many employees report high physical strain from overhead work and resulting musculoskeletal disorders. The consequences of these conditions extend far beyond everyday working life and can severely limit the quality of life of those affected. One solution to this problem may be the use of upper-limb exoskeletons, which are supposed to relieve the shoulder joint in particular. The aim of this literature review was to provide an overview of the use and efficacy of exoskeletons for upper extremities in the working environment.

**Methods:** A literature review was conducted using the PICO scheme and the PRISMA statement. To this end, a systematic search was performed in the PubMed, Web of Science and Scopus databases in May 2020 and updated in February 2022. The obtained studies were screened using previously defined inclusion and exclusion criteria and assessed for quality. Pertinent data were then extracted from the publications and analyzed with regard to type of exoskeleton used as well as efficacy of exoskeleton use.

**Results:** 35 suitable studies were included in the review. 18 different exoskeletons were examined. The majority of the exoskeletons only supported the shoulder joint and were used to assist individuals working at or above shoulder level. The main focus of the studies was the reduction of muscle activity in the shoulder area. Indeed, 16 studies showed a reduced activity in the deltoid and trapezius muscles after exoskeleton use. Kinematically, a deviation of the movement behavior could be determined in some models. In addition, study participants reported perceived reduction in exertion and discomfort.

**Discussion:** Exoskeletons for upper extremities may generate significant relief for the intended tasks, but the effects in the field (i.e., working environment) are less pronounced than in the laboratory setting. This may be due to the fact that not only overhead tasks but also secondary tasks have to be performed in the field. In addition, currently available exoskeletons do not seem to be suitable for all overhead workplaces and should always be assessed in the human-workplace context. Further studies in various settings are required that should also include more females and older people.

## Introduction

In many industrial working environments, the work load for employees is decreasing due to automated processes and use of robots. However, certain tasks and activities will continue to be performed manually in various professions such as in nursing and in the skilled trades, where a high degree of individuality, mobility and flexibility is important. And even in highly automated operations, e.g., in the automotive industry, human manual labor is essential for certain assembly steps and employees cannot (yet) be replaced by robots (Dengler and Matthes, 2018).

Although causes for musculoskeletal disorders (MSDs) are multifactorial (e.g., age, genetics, psychological factors), one of the main contributing factors is the biomechanical overload of the musculoskeletal system, which can be facilitated by regularly lifting heavy loads or performing monotonous repetitive work (Marras, 2005; da Costa et al., 2010). The shoulder joint with its large range of motion is particularly susceptible to injuries and overloads (Terry and Chopp, 2000). In Germany, for example, nearly 24% of employees are required to regularly carry heavy loads during work; and 16.9% of employees report working in forced positions (e.g., working overhead) on a regular basis (German Federal Ministry of Labour and Social Affairs, 2019). It is thus not surprising that one fifth of all sick leave days in German workplaces are due to MSDs. For workers over the age of 55 years, the frequency is even higher at 25%, resulting in overall annual production downtime costs of 85 billion euros (DAK Gesundheit, 2019; German Federal Ministry of Labour and Social Affairs, 2019).

To this end, many employers try to improve workplaces from an ergonomic point of view, e.g., through technical modifications, organizational changes (e.g., job rotation) or protective equipment (Berufsgenossenschaft Holz und Metall, 2017). Another promising approach may be the use of exoskeletons to reduce physical strain on employees (Steinhilber et al., 2018). Exoskeletons are not a new phenomenon; the first (military) prototypes emerged in the mid-1960s and they are becoming increasingly popular in medicine, for example in therapy or rehabilitation by supporting individuals in overcoming physical limitations (Bogue, 2015). Due to continued development of materials and control mechanisms, exoskeletons are now also being used in other fields (Bogue, 2018).

**FIGURE 1:**
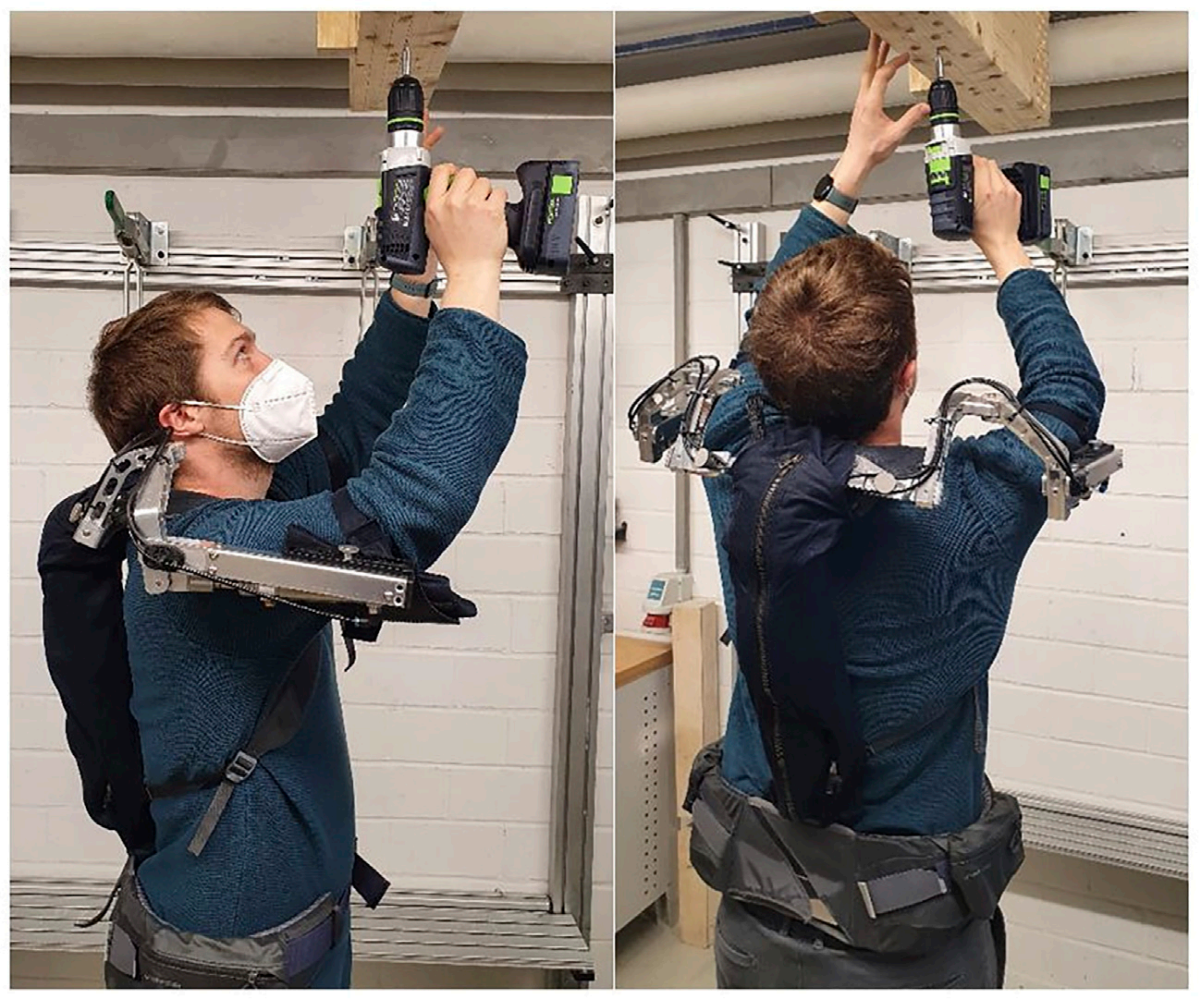
“Lucy” exoskeleton for over head work (e.g., Yao et al., 2021).

Exoskeletons can be classified according to their field of application (e.g., military, medical, industrial, sports); type of support (e.g., active, passive); or supported body segment (e.g., upper extremities, trunk/hip, lower extremities, full body, etc.). With regard to the working environment, most exoskeletons that are currently being developed and tested provide relief for the back, the hips and the shoulders (de Looze et al., 2015; Bogue, 2018). Due to the wide range of different exoskeletons and their different implications for both the user and the work process, this review will only focus on exoskeletons for the upper extremities. An example of this can be seen in Figure 1.

There is a wide variety of upper-limb exoskeletons with regard to construction forms and types of support. To date, the most common commercially available exoskeletons are shoulder-supporting exoskeletons that usually have a rigid structure on the back and arm cuffs, which are coupled (Exoskeleton Report, 2017). In addition, there are endpoint-based exoskeletons such as the Fortis exoskeleton, elbow-supporting exoskeletons, and whole-arm exoskeletons (Li and Lung Ng, 2018; Robo-Mate, 2020).

A review published in 2015 showed that many different exoskeleton types exist for industrial use (de Looze et al., 2015). Most upper extremity exoskeletons are anthropomorphic in design and support the entire arm or even the entire body. The review also revealed that exoskeletons can reduce muscle activity of the back muscles during lifting and static holding tasks, with active exoskeletons appearing to be more effective than passive ones. Main concerns regarding the use of exoskeletons in industrial workplaces pertain to wearing discomfort, high muscle activity of the loaded muscles after load redistribution and muscle deconditioning. Limitations of included research studies examining the use of exoskeletons were related to the setting, i.e., nearly all of the studies took place in a laboratory setting, hence no conclusion could be made on the use of exoskeletons in the field. In addition, most of the studies included only few participants, and considered muscle activity as main outcome parameter, with only few studies also examining biomechanical or other parameters (e.g., discomfort and acceptance).

Since 2015, the number of published studies on the use of upper-limb exoskeletons in the workplace has increased significantly, and there have also been significant changes to the design of these exoskeletons. Therefore, the aim of this literature review was to provide an updated overview of the current state of research on the use and efficacy of upper-limb exoskeletons in the working environment.

## Methods

We conducted a literature review based on the PRISMA Statement (Page et al., 2021) and systematically searched the PubMed, Web of Science und Scopus databases in May 2020, June 2021 and February 2022. The eligibility criteria were defined using the PICO framework (Richardson et al., 1995) as follows: 1) Participants/population: Although the target group of this review is workers/employees, we did not apply any exclusion criteria with regard to study population as many evaluation studies on exoskeletons used in workplaces were conducted in a laboratory setting with volunteers (e.g., students). Studies including participants with an existing disorder (e.g., lower back pain) were also included, since exoskeletons can be used to reintegrate persons with conditions of the musculoskeletal system into the workplace. 2) Intervention: We only included studies that evaluated upper-limb exoskeletons developed for or used in the workplace setting, and regardless of their mode of operation (active, passive, hybrid) or tasks for which they were designed (industry, care, handicraft, etc.). Studies that examined exoskeletons for other body parts such as back, legs or hands were not considered for this review. 3) Comparison and Outcome: No inclusion or exclusion criteria were defined for the comparison and outcome parameters. Studies were included that compared the respective exoskeleton with a “no exoskeleton situation”, as well as studies that investigated different exoskeletons simultaneously without a “no exoskeleton situation”. Furthermore, we only considered studies published in English or German before March of 2022. Preliminary studies and studies with less than seven participants were not included due to quality concerns. We used a combination of one or more of the following search terms: exoskelet* AND (active OR passive OR work* OR hybrid OR job OR occupation* OR lift* OR sit* OR stand* OR overhead OR bend* OR static* OR hold*OR manual OR handl*)

### Study Selection

**FIGURE 2:**
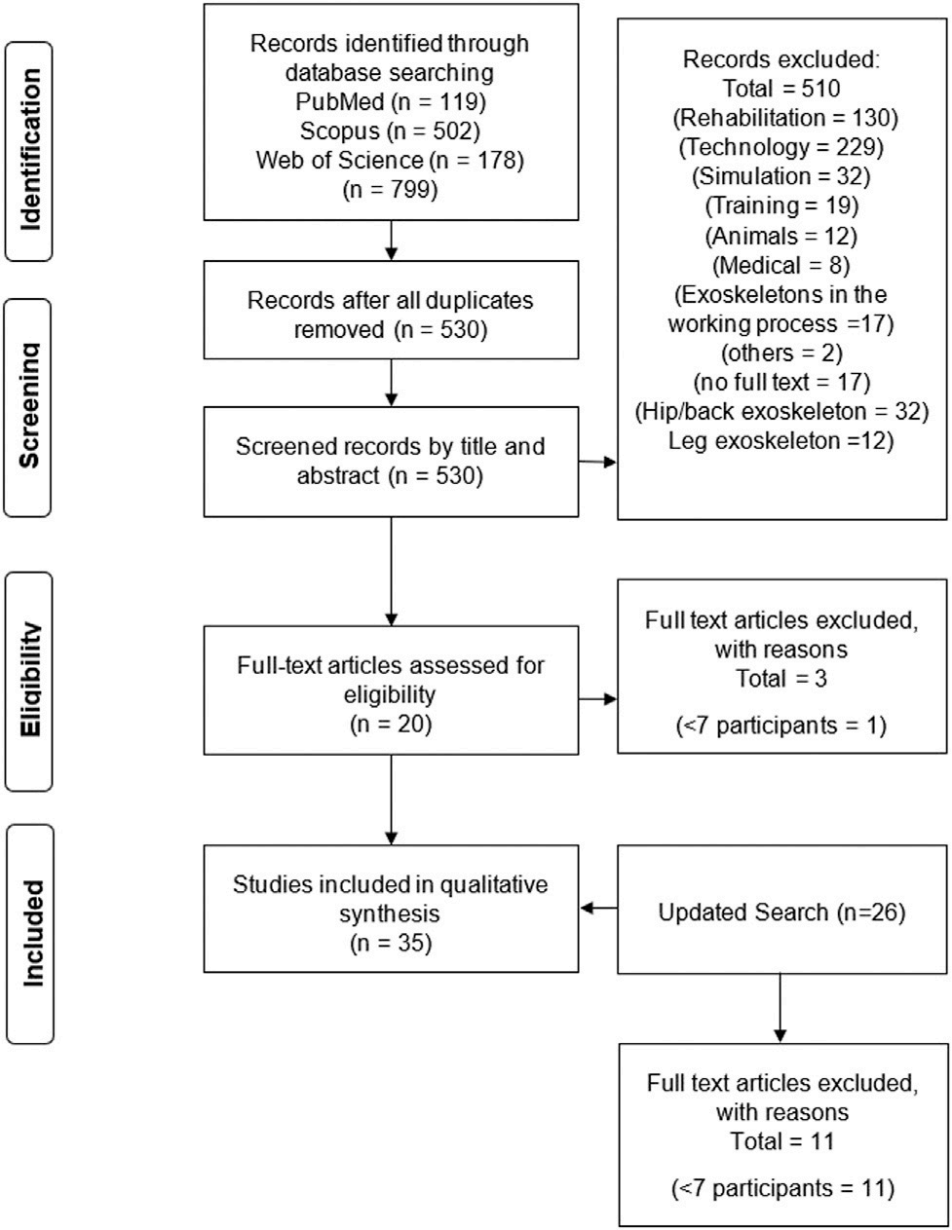
Flowchart of study section process (adapted according to Moher et al., 2009).

After the search was conducted, all duplicates were removed and the retrieved publications were screened for suitability based on their title. In a second step, the abstracts of all remaining studies were screened. In the final stage, we read all full texts of the remaining studies and used our aforementioned predefined inclusion and exclusion criteria to decide whether a study would be included in the review or not. The literature management process was done using the Citavi software (Version 6.3.0.0, Swiss Academic Software GmbH). For a flowchart of the study selection process, please refer to Figure 2.

### Data Extraction and Data Synthesis

All relevant data of the included studies were extracted and recorded by using a standardized data extraction form. The form included general information such as the authors’ names, publication date, title, study design, research question or objective, and study participants and their characteristics (e.g., numbers, sex, age). Furthermore, it contained information about the exoskeleton that was examined (e.g., model, version, power supply) and the study protocol (e.g., procedure, measurement setup, tasks, measurement instruments and outcome parameters). We also extracted information on the results of the study, the limitations as stated by the authors and further information such as funding source. After data collection, the data were synthesized. Attention was paid to the different tasks, forms of construction and support provided by the exoskeletons.

### Assessment of Methodological Quality

All included studies were assessed for quality by using a modified and adapted version of the National Heart, Lung, and Blood Institute (NHLBI) Quality Assessment Tool (QA) for Before-After (Pre-Post) Studies with No Control Group (National Heart, Lung and Blood Institute, 2021). This tool can be used to rate a study’s bias regarding research question, sample characteristics, description of the test procedure and measurement methods, and analysis of the data. It consists of 12 different items concerning: 1) study question; 2) participant eligibility criteria; 3) representativeness of sample; 4) inclusion of all eligible participants; 5) sample size; 6) intervention description; 7) outcome measures; 8) blinding; 9) loss to follow-up; 10) statistical methods; 11) multiple measures; and 12) group level analysis. However, for our purpose, we removed the item “blinding” as blinding is not possible in our included studies. Publications in which more than half of the items (6 or more) were answered with “no” were not considered further in this review due to poor quality.

## Results

The initial search in May 2020 yielded 119 publications from PubMed, 502 publications from Scopus and 178 publications from Web of Science databases. After removing the duplicates, 530 studies remained for the title and abstract screening. After this screening stage, 510 studies were excluded and 20 studies remained. The updated search in July 2021 and February 2022 resulted in 26 more publications. Eleven studies were excluded as they had less than seven study participants. No study was excluded due to poor quality. Thus, a final number of 35 studies was included in this review (Figure 1). The results of the individual studies can be seen in Table 1.

**TABLE 1:** Overview and brief description of included studies (▲ and ▼ indicate statistically significant higher/lower values compared to the execution without exoskeleton, unless specified otherwise; ▲ and ▼ indicate statistically not significant higher/lower value compared to the execution without exoskeleton, unless specified otherwise; OHW overhead work; RPE ratings of perceived exertion; RPD ratings of perceived discomfort; ROM range of motion; COP center of pressure; n.r. not reported; muscles: AD anterior deltoid; MD middle deltoid; PD posterior deltoid; IN infraspinatus; BB biceps brachii; BR brachioradialis; TB triceps brachii; ECR extensor carpi radialis; FCR flexor carpi radialis; ILL iliocostalis lumborum pars lumborum; LD Latissimus dorsi; RH rhomboids; TP trapezius; PM pectoralis major; SE serratus anterior; RA rectus abdominis; OE obliquus externus abdominis; TBA tibialis anterior.

Author (year)	Exoskeleton	Subjects	Tasks	Muscle Activity (mean)	Kinematics/kinetics	Other effects
Alabdulkarim and Nussbaum (2019)	Fortis; ShoulderX; Fawcett Exovest (all passive)	16 (8♂, 8 ♀)	OHW	▼ AD + MD (ShoulderX)	—	▼ Maximum acceptable frequency (Fortis, ♀)
				▲ ILL (Fortis)		▲ Errors (Fortis)
		age: 23.0 ± 2.1		▲ AD + MD (Fortis, Exovest)		▼ RPD in lower back (ShoulderX, ♂)
						▲ RPD in thigh (Fortis, ♂)
Blanco et al. (2019)	ExIF project upper limb exoskeleton (active)	10 (8♂, 2♀)	Holding arm on shoulder level	▼ PM + RH (with load)	—	—
		age: 28.8 ± 3.4				
Blanco et al. (2020a)	ExIF project upper limb exoskeleton (active)	10 (5♂, 5♀)	Working on shoulder level	—	▼ Movement speed	—
					▼ Movement accuracy	
		age: 29.8 ± 6.8			▼ Dispersion in the movement	
Blanco et al. (2020b)	ExIF project upper limb exoskeleton (active)	12 (11♂, 1♀)	Lifting and holding arm at shoulder level	—	—	▼ Oxygen consumption
						▼ Standard deviation of the time spent in the upward motion
		age: 27.6 ± 5.5				
Blanco et al. (2022)	ExIF project upper limb exoskeleton (active)	12 (11♂, 1♀)	Lifting and holding arm at shoulder level	▼ BB (up to 64%) + TB (up to 37%) + RH (up to 40%) + PM (up to 38%) (load + no load)	—	▼ HR
		age: 27.6 ± 5.5				
Daratany and Taveira (2020)	EksoBionic EksoVest (passive)	12 (12♂)	OHW	—	—	▼ Heart rate (3–18%)
						- Usefulness (4-5.5) on 7-point scale
Desbrosses et al. (2021)	EXHAUSS Stronger exoskeleton; Skelex (all passive)	29 (15♂, 14♀)	Lifting and holding arm at shoulder level	▼ AD + TP + BB + ECR (both, 2 kg +8 kg)	▲ antero-posteriore amplitude COP (EXHAUSS, 2 kg)	▼RPE Upper Limb and lower back (EXHAUSS)
					▼antero-posteriore amplitude COP (EXHAUSS, 8 kg)	
		age: ♂23 ± 3			▼ Total length COP (both exo 8 kg; Skelex 2 kg)	
		♀22 ± 2				
Ferreira et al. (2020)	Skelex (passive)	88	Field: >30% OHW 6 Workstations (WS) (4 weeks)	—	—	**Help performing**
						main task: - 0% (WS 5) up to 87% (WS 1)
						secondary task: - 2% (WS 4) up to 74% (WS 1)
						**Intention to use**
						initial: - 25% (WS 5) up to 83% (WS 2)
						final: - 10% (WS 4) up to 53% (WS 1)
Grazi et al. (2020)	H-PULSE exoskeleton (semi-passive)	10 (10♂)	OHW	▼ AD (up to 42%) + PD (up to 42%) + TP (up to 50%)	—	▼ Heart rate (up to 10%)
						▼ RPE
		age: 28.5 ± 2.5				
Groos et al. (2022a)	Airframe (passive)	20 (9♂, 11♀)	OHW	▼ AD + MD + TP	—	- stress-reducing effect (shoulder, upper arm, lower back)
		age: 31.9 ± 13.4				
Hefferle et al. (2021)	Crimson Dynamics; Skelex V1 (all passive)	8 (8♂)	Field: OHW	—	—	▼RPE neck (Skelex)
						▼RPE shoulders (Crimson Dynamics)
		age: 37,5 ± 13,0				
						▼RPE spine (both)
						▼RPE whole body (Crimson Dynamics)
						▼RPE whole body (Skelex)
Huysamen et al. (2018)	Robo.Mate (passive)	8 (4♂, 4♀)age: 38 ± 10	Lifting and holding arm at shoulder level with different weights	▼ BB (49%, 2 kg) + MD (62%, 2 kg) + RA (13%, 0 kg)	—	▼ RPE (41 %)
						- No increased perceived pressure
						- Half of the participants rate the exoskeleton as acceptable
Iranzo et al. (2020)	Airframe (passive)	12 (11♂, 1♀)	Field: OHW	▼ AD (34 %)	- Limitations of the ROM by a maximum of 5°	—
				▼ TP (21 %)		
		age: 35 ± 5				
Kim et al. (2021)	EksoVest; (passive)	Exo Group 41 (30♂, 3♀, 8 n.r.)	Field: OHW (18 months)	—	—	- no significant differences in perceived work intensity
						- no significant differences in RPD
		age: 38				
		Control group				
		83 (47♂, 14♀, 22 n.r.)				
		age: 38				
Maurice et al. (2020)	Paexo Shoulder (passive)	12 (12♂)	OHW	▼ AD (54 %)	▼ COP velocity (14%)	▼ Oxygen consumption (33 %)
						▼ Heart rate (19 %)
		age: 23.2 ± 1.2			▲ shoulder abduction, ▲shoulder flexion and shoulder rotation (only for the start position)	▼ RPE (21 %)
						- Subjective restriction of movement in extreme positions
						- No effect on movement duration
McFarland et al. (2022)	Airframe (passive)	12 (12♀)age: 20 ± 1.8	OHW + work at shoulder level	—	▲ minimum shoulder elevation (35–36%, support mode: 1.81 kg and 2.72 kg)	▼ RPE
					▼shoulder axial rotation angle by 67.0° (316%, support mode: 0,91 kg)	▼ RPD (right shoulder, right elbow)
					▲mean forearm pronation by 22.6° (62.3% support mode: 0,91 kg)	- no difference in task duration
Moyon et al. (2018)	Skelex (passive)	9 (5♂, 4♀)	Field: OHW	—	—	▼ Heart rate (13.5 %)
		age: 20-46				
Nassour et al. (2021)	Carry (active)	12 (12♂)	Holding and Carrying Weights	▼ BB (35%) + BR (37%) + FCR (24%) + TP (25%) (Carrying and Holding)	▲mean maximum elbow flexion moment in post-test observation (long holding +carrying)	▼ Metabolic rate long holding (61%)
		age: 32.2 ± 7.8				▼ Metabolic rate carrying (32%)
Otten et al. (2018)	Lucy (active)	8	OHW	▼ AD (58 %)	—	▼RPE
						▲ Perceived support with full exoskeleton support
Pacifico et al. (2020)	Proto-Mate (passive)	15 (11♂, 4♀)	OHW; Reaching test; holding arm at shoulder level	▼ AD + MD + TP + PM (OHW)	▼ ROM (shoulder abduction– adduction; elbow)	—
				▼ AD (36 %) + PM (42 %) (reaching)		
		age: 32 ± 9				
				▲ PD (20 %) (reaching)		
				▼ AD + MD + TP + TB + PM + LD (holding)		
Pacifico et al. (2022)	Mate (passive)	7 (7♂)age: 40 ± 14	Lab: OHW	▼ AD + MD +TP (field, mounting	—	▼ RPE (shoulder, arm and lower back, in field and lab)
				▼ AD + MD + TB (field, Dismounting		- global usability score (60–87%)
				▼ AD + MD + TP + PM + TB + PD (lab)		- global acceptance score (61–79%)
			Field: Mounting and dismounting panels in overhead height (10–25 kg)			
Perez Luque et al. (2020)	EksoVest; Paexo; Mate (all passive)	17 (11♂, 6♀)age: 25 (18-46)	OHW	—	- Mate deviates most from the optimal movement in the shoulder joint	- Subjects’ preferences:
						1. Paexo (12)
						2. EksoVest (9)
						3. Mate (0)

Pinho et al. (2020)	ShoulderX (passive)	7	OHW, work on/under shoulder level	▼ AD + MD (OHW, elbow on shoulder level)	—	—
				▼ AD (work on shoulder level)		
Pinto et al. (2021)	Mate (passive)	12	Lifting and holding arm at shoulder level	▼ AD + MD + TP	—	—
		age: (20–30)				
Rashedi et al. (2014)	WADE (passive)	12 (12♂)	Lifting and holding arm atshoulder level	▼ AD (50 %)	—	▼ RPD (shoulder up to 57%; upper arm up to 45%)
				▲ ILL (31-88 %)		
		age: 27.0 ± 2.6				
Schmalz et al. (2019)	Paexo shoulder (passive)	12 (6♂, 6♀)	OHW	▼ AD + MD + PD + BB + TP + LD + SE + OE (22 %-61 %)	▲ mean shoulder abduction and elbow flexion	▼ Heart rate (5%)
						▼ Oxygen consumption (12%)
		age: 24 ± 3				
Smets (2019)	EksoVest (passive)	10 (9♂, 1♀)	Field: OHW (3 month)	—	- Restrictions in non-neutral trunk posture	▼ RPD in arms, back and neck
		age: 45 (20-62)			- No relevant movement restrictions	▲ Subjective task performance
						- No thermal discomfort
Spada et al. (2018)	IUVO (passive)	18 (18♂)	holding arm at and work shoulder level	—	—	▲ Holding time (56%)
						▲ Precision
		age: 43.0 ± 11.1				▼ RPE
Spada et al. (2017)	Airframe (passive)	31 (31♂)	holding arm and work at shoulder level	—	—	▲ Holding time (31%)
						▲ Precision (16.7%)
		age: 51.5 ± 4.7				
Sylla et al. (2014)	ABLE (active)	8	OHW	—	- slight modifications of arms movements	▲ Execution time by 1 s
		age: 24 ± 7				
Theurel et al. (2018)	EXHAUSS Stronger exoskeleton (passive)	8 (4♂,4♀)	Lifting, carrying and stacking weights	Lifting	Lifting	▼ RPE (carrying)
				▼ AD	▼ shoulder flexion and external rotation angles	▲ Time required for stacking
		age:		▲ TB + TBA Carrying	▲ elbow flexion angle	▲ Cardiac cost (lifting)
		♂ 31 ± 2		▼ TB Stacking	▲ maximal oscillation in antero-posterior	
		♀ 33 ± 3		▼AD	Carrying	
					▲ averaged flexion angle (elbow)	
					+ averaged abduction angle (shoulder)	
					Stacking	
					▼ averaged flexion angle (elbow)	
					+ averaged abduction angle (shoulder)	
Van Engelhoven et al. (2018)	ShoulderX (passive)	13 (13♂)	OHW	▼ AD (up to 64%) + TP (up	—	—
				to 46%) + IN (up to 24 %)		
		age: 37 ± 13		▲ TB (up to 4 %)		
Vries et al. (2021)	Skelex 360 (passive)	11 (11♂)	OHW	All tasks:		▼ RPE
				▼ MD + TP + BB		
		age: 36.2 ± 8.4		Nearly all tasks:		
				▼ AD + TP		
Wang et al. (2021)	Not named (passive)	**Lab:**	Lab: simulated fruit thinning and pesticide spraying Field: fruit thinning and pesticide spraying	▼AD (only Group A, pesticide spraying) ▼ AD (Group B, all conditions and group A fruit thinning ▼ AD + MD+ PD (Group C+D)	- no changes in the lifting angel of the upper limb	▼ RPE (lab)▼ RPE (field)
		A: 8 (8♂)				
		age: 30.3 ±5.3 B: 10 (10♂) age: 50.3 ±9.0				
		**Field:** C: 4 (4♂) age: 50.5 ±6.3 D: 3 (3♂, 1♀)age: 53.0 ±12.0				
Weston et al. (2022)	EksoVest; Airframe; ShoulderX all passive)	12 (6♂,6♀)age: ♂ 21.2 ± 2.9 ♀ 22.5 ± 3.3	—	—	▼ resultant spinal loads (only ShoulderX)	▼ tissue saturation index (only shoulderX)
						▲ RPD shoulder + upper arm (ShoulderX in comparison to EksoVest + Airframe)
						▼ RPD upper arm (only EksoVest)
						▼ RPD wrist/hand

### Study Characteristics

All studies were published between 2014 and 2022, with the majority of studies (*n* = 32) published within the last 4 years (2018 and later). All studies can be considered quasi-experimental studies: 27 were conducted in the laboratory setting, six in the field setting (i.e., workplace environment), and two studies (Wang et al., 2021; Page et al., 2021) used both settings. The data collection duration of the field studies was less than one working day in four studies. In one study, the exoskeleton was worn for 4 weeks (max. 2 h per day) and in another study, the wearing time was 3 months (average 7.7 h per day). Only one field study (Kim et al., 2021) examined the exoskeleton for 18 months.

A total of 636 participants were included in the 35 studies (ranges *n* = 7–124). 59.1% of participants were male (*n* = 376), 17% were female (*n* = 108) and no gender information was provided for 24.1% of participants (*n* = 153). The average age of participants ranged between 20 and 51 years; it was above 40 years in five studies (Spada et al., 2017; Spada et al., 2018; Smets 2019; Wang et al., 2021; Page et al., 2021) and above 50 years in one study (Spada et al., 2017). In four studies (Otten et al., 2018; Daratany and Taveira 2020; Ferreira et al., 2020; Pinho et al., 2020), the age of participants (*n* = 111) was not reported. In almost all studies, participants were described as healthy, or as having been free of symptoms of MSDs in general or for certain areas and time periods. Six studies (Sylla et al., 2014; Moyon et al., 2018; Otten et al., 2018; van Engelhoven et al., 2018; Blanco et al., 2020a; Kim et al., 2021) did not provide any information on the health status of participants.

Ten studies were conducted or supported by industrial companies that can be considered users of exoskeletons. Of these, seven were car manufacturers, one was an aircraft manufacturer and one was a manufacturer of drywall systems. In addition, six studies were conducted or supported by manufacturers of exoskeletons.

### Exoskeleton Design, Task Descriptions, Testing Methods and Data Collection

In the 35 studies included in this review, 18 different exoskeletons were evaluated. The most frequently evaluated exoskeletons were Skelex (Skelex, *n* = 5), Airframe (Levitate, *n* = 5), EksoVest (EksoBionics, *n* = 5), Mate (IUVO, *n* = 5), an active upper-limb exoskeleton from the ExIF Project ((Blanco et al., 2020b), *n* = 4), ShoulderX (SuitX, *n* = 4) and Paexo (OttoBock, *n* = 3). 72% of exoskeletons were passively operated. Only four models were active exoskeletons, and one exoskeleton (Grazi et al., 2020) was reported to be semi-passive. Most exoskeletons (*n* = 11) only supported the shoulder joint. Three exoskeletons (Rashedi et al., 2014; Alabdulkarim and Nussbaum, 2019) were endpoint based, i.e., they are attached to the upper body with a strap, but the end is directly connected to the tool or material. Three exoskeletons (Sylla et al., 2014; Huysamen et al., 2018; Blanco et al., 2019; Blanco et al., 2020a; Blanco et al., 2020b; Blanco et al., 2022) supported both the shoulder and elbow joints and one exoskeleton (Nassour et al., 2021) only supported the elbow joint.

In most studies (*n* = 31), the use of the exoskeleton (no exo/with exo) was regarded as an independent variable. Nine studies additionally included different weights or support settings of the exoskeletons. In five studies, different models were compared in addition to the first condition (no exo/with exo). Furthermore, in three other studies, different support modes of one exoskeleton were compared.

The studies examined two different types of tasks, i.e., static and dynamic tasks. The static tasks included lifting and holding the arm at overhead level, and lifting and holding the arm at shoulder level. Dynamic tasks included working above the head, working at shoulder level and carrying/transporting objects. Furthermore, all work-related tasks carried out in the field studies were dynamic tasks. Although the tasks in the field settings varied more as compared to the laboratory setting, the main focus of the workplaces used in the studies was always on overhead work (OHW). Six of the field studies were conducted in automotive assembly plant and one in a fruit orchard.

Most studies (*n* = 21) examined the exoskeleton based on muscle activity of the involved muscles in the shoulder, arm and upper body. Furthermore, kinematic parameters (*n* = 12) such as the range of motion (ROM), joint angles trajectories and body segments speed were used for evaluation. Other objective parameters were heart rate during task (*n* = 5), postural balance (*n* = 3), time taken to complete a task (*n* = 3), energy expenditure during task (*n* = 2) and number of repetitions completed (*n* = 2). The main subjective parameters used in the studies were ratings of perceived exertion (RPE, *n* = 16), usefulness (*n* = 5) and ratings of perceived discomfort (RPD, *n* = 9).

### Effects of Exoskeleton Use on Muscle Activity

For OHW in the laboratory setting, most exoskeletons lead to a reduction of the activity of the anterior and middle deltoid in study participants (Otten et al., 2018; van Engelhoven et al., 2018; Alabdulkarim and Nussbaum, 2019; Schmalz et al., 2019; Grazi et al., 2020; Maurice et al., 2020; Pacifico et al., 2020; Pinho et al., 2020; Vries et al., 2021; Wang et al., 2021; Groos et al., 2022a; Page et al., 2021). Only two exoskeletons showed a significantly higher muscle activity in anterior and middle deltoid in OHW. Furthermore, use of the Fortis exoskeleton resulted in a higher activity in the iliocostalis lumborum pars lumborum (Alabdulkarim and Nussbaum, 2019). Three studies also showed a reduction of the activity of the posterior deltoids (Schmalz et al., 2019; Grazi et al., 2020; Wang et al., 2021; Page et al., 2021). The trapezius muscle was also found to be less active in study participants when using an exoskeleton (Schmalz et al., 2019; Grazi et al., 2020; Pacifico et al., 2020; Vries et al., 2021; Groos et al., 2022b; Page et al., 2021). Only one study revealed an increased muscle activity in OHW, namely in the triceps brachii (van Engelhoven et al., 2018).

For static holding at shoulder level, reduced deltoid (anterior and middle) activity was found (Rashedi et al., 2014; Huysamen et al., 2018; Theurel et al., 2018; Pacifico et al., 2020; Desbrosses et al., 2021; Pinto et al., 2021). In addition, the use of an exoskeleton decreased the activity of the pectoralis major (Blanco et al., 2019; Pacifico et al., 2020; Blanco et al., 2022), of the biceps brachii (Huysamen et al., 2018; Desbrosses et al., 2021; Blanco et al., 2022) and of the triceps brachii (Desbrosses et al., 2021; Pinto et al., 2021; Blanco et al., 2022). In only two studies the muscle activity (iliocostalis lumborum pars lumborum and triceps brachii) increased as a result of wearing an exoskeleton (Rashedi et al., 2014; Theurel et al., 2018).

In the field setting, only three studies investigated muscle activity among study participants who wore an exoskeleton, and two found a significantly reduced activity of the anterior deltoid (Iranzo et al., 2020; Wang et al., 2021). Iranzo et al. (2020) also showed a significant reduced activity of the trapezius muscle. Furthermore, Pacifico et al. (2020) reported a reduced activity of the middle and anterior deltoid, the triceps brachii and the trapezius muscle, but none of these reductions was statistically significant.

Two studies also investigated muscle activity of study participants when carrying objects with suitable exoskeletons with elbow joint support. The investigators reported that the use of an exoskeleton reduce the activity of the triceps brachii (Theurel et al., 2018), biceps brachii, trapezius, brachioradialis and flexor carpi radialis (Nassour et al., 2021).

### Kinematic and Kinetic Effects of Exoskeleton Use

Kinematically, an increased shoulder abduction and an increased shoulder flexion could be found during the OHW for the Paexo Shoulder exoskeleton, (Schmalz et al., 2019; Maurice et al., 2020), whereby Maurice et al. (2020) found an increased shoulder flexion only in the start pose. Also, the Centre of Pressure (COP) velocity increased during OHW (Maurice et al., 2020). For the Proto-Mate exoskeleton, Pacifico et al. (2020) observed a decreased ROM for both the shoulder (abduction-adduction) and the elbow joint. By using the Airframe Exoskeleton, McFarland et al. (2022) reported a higher minimum shoulder elevation, a higher mean forearm pronation and a lower shoulder axial rotation angel. Furthermore, when comparing three different exoskeletons (ExoVest, Paexo Shoulder, Mate), the movement while wearing the Mate exoskeleton deviates most from the movement without exoskeleton in the shoulder joint (Perez Luque et al., 2020).

For static holding tasks, Desbrosses et al. (2021) found an increased antero-posterior amplitude of COP for the EXHAUSS exoskeleton when the participants were holding a light weight (2 kg), as well as a decreased antero-posterior amplitude of COP when the participants were holding a heavy weight (8 kg). In addition, the total length of the COP was significantly reduced when using the EXHAUSS exoskeleton (8 kg) and the Skelex (2 kg; 8 kg) exoskeleton.

In a field setting, Iranzo et al. (2020) kinematically detected only a 5° reduction in ROM and Smets (2019) identified restrictions in non-neutral trunk posture.

For carrying weights, two studies showed an increased flexion angle (elbow) and abduction angle (shoulder) (Theurel et al., 2018). Furthermore, the mean maximum elbow flexion moment in the post-test observation increased when using an active elbow joint supporting exoskeleton (Nassour et al., 2021).

### Effects of Exoskeleton Use on Other Objective Parameters

Five studies revealed a reduction in heart rate for OHW in participants who wore an exoskeleton in the laboratory setting (Schmalz et al., 2019; Daratany and Taveira 2020; Grazi et al., 2020; Maurice et al., 2020; Blanco et al., 2022). Similar results were observed in the field setting (Moyon et al., 2018), albeit the effects did not reach statistical significance. In addition, three studies showed that the use of an exoskeleton reduces oxygen uptake during OHW (Schmalz et al., 2019; Maurice et al., 2020) and in a static posture (Blanco et al., 2020b). For shoulder level work, Spada et al. (2018) found that both the IUVO exoskeleton and the Levitate increased hold time by 56 and 31% respectively, with more precise work. In addition, Nassour et al. (2021) showed that when using the active, elbow supporting exoskeleton, the metabolic rate during carrying and holding was reduced by 32 and 61%, respectively. However, the execution time of a task increased by one second with the active exoskeleton “ABLE” (Sylla et al., 2014). McFarland et al. (2022) did not find a longer task duration for the passive exoskeleton Airframe. Furthermore, one study (Weston et al., 2022) found lower values for the tissue saturation index in the shoulder muscles with the ShoulderX exoskeleton compared to task execution without an exoskeleton.

### Effects of Exoskeleton Use on Subjective Parameters

Reduced RPE was reported for OHW in both the laboratory and field settings, as well as during static tasks (Huysamen et al., 2018; Otten et al., 2018; Spada et al., 2018; Grazi et al., 2020; Maurice et al., 2020; Desbrosses et al., 2021; Hefferle et al., 2021; Vries et al., 2021; Wang et al., 2021; Groos et al., 2022a; McFarland et al., 2022). Furthermore, RPD among study participants decreased when using an exoskeleton in OHW, static tasks in laboratory setting (Rashedi et al., 2014; Huysamen et al., 2018; Alabdulkarim and Nussbaum 2019; Smets 2019; McFarland et al., 2022; Weston et al., 2022). Only Alabdulkarim and Nussbaum (2019) showed an increased RPD for the Fortis exoskeleton in male participants at the thigh.

Furthermore, when comparing three different exoskeletons (EksoVest, Paexo Shoulder, Mate), 12 participants rated the Paexo and nine participants the ExoVest as the best supporting system for OHW. No participant voted in favour of the Mate Exoskeleton (Perez Luque et al., 2020). In another laboratory study (Daratany and Taveira 2020), the participants rated the usefulness of the exoskeleton at the overhead workstation (WS) after 8 min of use with 4–5.5 points on a 7-point scale. A second comparison study showed that the RPD in the shoulder joint is higher when using the ShoulderX exoskeleton as compared to using EksoVest or the Airframe exoskeleton (Weston et al., 2022).

In a 4-week field test, Ferreira et al. (2020) investigated the usefulness of exoskeletons at primarily overhead workstations on a daily basis and participants’ intention to use them before and after the test. Depending on the WS, the usefulness varied between 0 (WS 5) and 87% (WS 1) for the main task, and between 2 (WS 4) and 74% (WS 1) for the secondary task. Furthermore, the study showed that the intention to use exoskeletons decreased from an initial range between 25% (WS 5) and 83% (WS 2) to a post-test range between 10% (WS 4) and 53% (WS 1) within 4 weeks. However, Kim et al. (2021) did not find any significant reduction in either RPD or perceived work intensity compared to the control group in an 18-month field study.

### Quality Assessment

All studies included in this review had at least moderate study quality (Table 2), and only minor biases can be assumed. The average quality score was 8.02 out of 11 possible points. Only two studies (van Engelhoven et al., 2018; Iranzo et al., 2020) scored 10 points, and 4 studies (Sylla et al., 2014; Daratany and Taveira 2020; Grazi et al., 2020; Page et al., 2021) scored six points. The most common limitation of the studies was that the measurements were only carried out once. Repeated performance and measurements would have increased reliability and validity of the results.

**TABLE 2:** Results of the quality assessment. The reader is referred to National Heart, Lung and Blood Institute (2021) for further details on the different items of the quality assessment tool.

Author (year)/Item	1	2	3	4	5	6	7	8	9	10	11	12	Sum
Alabdulkarim and Nussbaum (2019)	1	1	1	1	1	1	1	0	0	1	0	1	**9**
Blanco et al. (2019)	1	0	1	1	1	1	1	0	0	1	0	1	**8**
Blanco et al. (2020a)	1	1	1	1	1	1	1	0	0	1	0	0	**8**
Blanco et al. (2020b)	1	1	1	1	1	1	1	0	0	1	0	0	**8**
Blanco et al. (2022)	1	1	1	1	1	1	1	0	0	1	0	0	**8**
Daratany and Taveira (2020)	1	1	0	1	1	1	1	0	0	0	0	0	**6**
Desbrosses et al. (2021)	1	1	1	1	1	1	1	0	0	1	1	0	**9**
Ferreira et al. (2020)	1	1	1	1	1	1	1	0	0	1	0	0	**8**
Grazi et al. (2020)	1	0	1	0	1	1	1	0	0	1	0	0	**6**
Groos et al. (2022b)	1	1	1	1	1	1	1	0	0	0	0	0	**7**
Hefferle et al. (2021)	1	1	1	1	1	1	1	0	0	1	0	0	**8**
Huysamen et al. (2018)	1	1	1	1	1	1	1	0	0	1	1	0	**9**
Iranzo et al. (2020)	1	1	1	1	1	1	1	0	0	1	1	1	**10**
Kim et al. (2021)	1	1	1	1	1	1	1	0	0	1	0	1	**9**
Maurice et al. (2020)	1	1	1	1	1	1	1	0	0	1	0	1	**9**
McFarland et al. (2022)	1	1	1	1	1	1	1	0	0	1	0	1	**9**
Moyon et al. (2018)	1	1	1	1	1	1	1	0	0	1	0	0	**8**
Nassour et al. (2021)	1	1	1	1	1	1	1	0	0	1	0	0	**8**
Otten et al. (2018)	1	0	1	1	1	1	1	0	0	1	0	0	**7**
Pacifico et al. (2020)	1	1	1	1	1	1	1	0	0	1	1	0	**9**
Pacifico et al. (2020)	1	1	1	1	0	1	1	0	0	0	0	0	**6**
Perez Luque et al. (2020)	1	1	1	1	1	1	1	0	0	1	0	0	**8**
Pinho et al. (2020)	1	1	1	1	1	1	1	0	0	1	0	0	**8**
Pinto et al. (2021)	1	0	1	0	1	1	1	0	0	1	1	0	**7**
Rashedi et al. (2014)	1	1	1	1	1	1	1	0	0	1	0	0	**8**
Schmalz et al. (2019)	1	0	1	1	1	1	1	0	0	1	1	0	**8**
Smets (2019)	1	1	1	1	1	1	1	0	1	0	1	0	**9**
Spada et al. (2018)	1	1	1	1	1	1	1	0	0	0	0	0	**7**
Spada et al. (2017)	1	1	1	1	1	1	1	0	0	1	0	0	**8**
Sylla et al. (2014)	1	0	1	1	1	1	1	0	0	0	0	0	**6**
Theurel et al. (2018)	1	1	1	1	0	1	1	0	0	1	1	0	**8**
van Engelhoven et al. (2018)	1	1	1	1	1	1	1	0	0	1	1	1	**10**
Vries et al. (2021)	1	1	1	1	1	1	1	0	0	1	0	0	**8**
Wang et al. (2021)	0	1	1	1	1	1	1	0	1	1	1	0	**9**
Weston et al. (2022)	1	1	1	1	1	1	1	0	0	1	0	0	**8**

## Discussion

The aim of this review was to provide an updated overview of the current state of research on the use and efficacy of upper-limb exoskeletons in the working environment. To this end, we conducted a review to identify relevant studies on upper-limb exoskeletons designed for the working process. We included a total of 35 studies, whereby most (*n* = 32) were published after 2017. In addition, ten studies were conducted or supported by industrial companies, which underlines the economic and scientific importance of this topic. Compared to a previous review published in 2015 (de Looze et al., 2015), it can be shown that mainly exoskeletons with rather limited functionality and complexity (i.e., passive, shoulder support only) are established on the market.

Most studies included in our review demonstrated that upper limb exoskeletons are objectively and subjectively effective in reducing user stress in OHW, although there are differences between studies conducted in laboratory and field settings. Furthermore, we observed that some exoskeletons still are less accepted among users, but the reasons for this lower acceptance are multifactorial and must always be considered in the context of workplace, user and exoskeleton when used. In addition, the studies have some limitations with regard to both design and conduct that need to be considered in future research.

### Objective and Subjective Efficacy in Laboratory and Field Setting

Almost all exoskeletons reduce the study participants’ activity of the target muscles (anterior, middle and posterior deltoid and trapezius) for tasks on shoulder level and OHW, especially in the laboratory setting. They can therefore be considered effective in relation to their main task and relieve the shoulder joint. According to Schmalz et al. (2019) and Pacifico et al. (2020), a reduction in shoulder compression forces can be achieved from reduced activity of the shoulder girdle muscles, which in turn can have a positive effect on the health of the glenohumeral joint and the surrounding tissues (e.g., tendons, capsule). The exception to this seems to be endpoint-based exoskeletons such as the Fortis Exoskeleton and the Fawcett Exovest, where OHW increases the muscle activity of anterior and middle deltoid. Other muscles such as biceps brachii, pectoralis major and the latissimus dorsi muscle are also relieved through use. Only in the iliocostalis lumborum pars lumborum was an increased muscle activity found in OHW in two studies, thus it can be assumed that the load redistribution through the exoskeleton only leads to an increased load in other body regions to a small extent. Furthermore, a consistently reduced heart rate and oxygen consumption as well as an improved performance (e.g., longer maximum holding time) also provide preliminary evidence for the effectiveness of the exoskeletons in decreasing physical strain among participants. In addition, exoskeletons appear to be suitable for precision tasks in OHW at the workplace. Due to the support of the upper arms on the connecting shells and the reduced muscular fatigue, persons who regularly carry out such tasks may benefit from wearing an exoskeleton. Subjectively, a reduction of RPE and RPD could be observed, which in turn may lead to increased acceptance.

Although some studies in the workplace setting provided first evidence of a reduction in the employees’ workload (muscle activity, heart rate, RPE), the effects did not always reach statistical significance. This could be due to the fact that the respective exoskeletons are primarily optimized for their actual application purpose and peripheral tasks, which are unavoidable in the field, are not supported or even disrupted. However, the longest field study included in this review (Kim et al., 2021) failed to show any effects on RPD and perceived work intensity compared to a control group; thus, the EksoVest exoskeleton did not lead to a subjective advantage.

Furthermore, our review has shown that exoskeletons also appear to be effective for transporting/carrying objects, i.e. they significantly reduced the muscle activity of triceps brachii and the metabolic rate. However, only two of the included studies examined exoskeletons for transporting/carrying objects, evidence is thus limited and further research is needed.

Kinematically, most models showed good agreement with the movement without exoskeleton with the respective users. In some models, the ROM was slightly reduced; it can thus be assumed that the exoskeletons do not reproduce the full ROM of the human being, especially in extreme positions. For the Paexo exoskeleton, two studies showed increased shoulder abduction angles and elbow flexion angles during use, as well as greater shoulder flexion and rotation in the neutral zero position. In addition, regarding the Airframe exoskeleton, one study (McFarland et al., 2022) showed a lower shoulder axial rotation angle and a higher mean forearm pronation as well as a higher minimum shoulder elevation.

### Acceptance of and Familiarisation With Upper-Limb Exoskeletons

In a further comparison (Perez Luque et al., 2020) with three models, users preferred exoskeletons that had the least kinematic deviation from movement without an exoskeleton. In contrast, an exoskeleton with the greatest deviation from normal movement had the lowest acceptance rate among users. Thus, one may conclude that the more natural a movement feels when using an exoskeleton (i.e., high correlation between movement with and without exoskeleton), the higher the acceptance among the users. However, this study did not have a structured familiarization phase.

In general, most studies only had a short wearing period and brief familiarization period. Future research should apply longer periods of familiarization as movements generated by the exoskeleton likely disturb the user. It is thus preferable that the user first familiarizes him-/herself with these new tasks. Since human movement is usually very efficient, wearing an exoskeleton without proper familiarization could lead to a more inefficient movement overall and may facilitate fatigue of the user. However, an adaptation is possible through learning effects, and it is possible that the user’s movement can adapt to the support provided by an exoskeleton when worn over a longer time period.

Ferreira et al. (2020) were also able to show that in different workplaces with at least 30% OHW, the perceived usefulness and the intention to use an exoskeleton can differ greatly. The tested exoskeleton showed the highest usefulness and acceptance in high OHW tasks and long static tasks at the same time. However, prolonged OHW can also lead to various acute physiological adaptations such as reduced blood circulation, which in turn may induce other negative consequences such as local muscle fatigue, higher heart rate and higher blood pressure (Grieve and Dickerson 2008). The extent to which these symptoms also manifest when using overhead exoskeletons needs to be further investigated; however, from an ergonomic perspective, long static tasks at the workplace should be avoided and varied/dynamic workplaces should be designed instead. In the future, hybrid systems with appropriate sensors could adapt the degree of support of an exoskeleton to the movement and thus lead to higher efficiency.

It should also be noted that in one study (Ferreira et al., 2020), the intention to use the exoskeleton decreased at different workstations over a period of 4 weeks. Hensel and Keil (2019) reported similar results for exoskeletons used to support the lower back. This could be due to various reasons such as discomfort, heat, insufficient perceived support, unsuitability for the workplace or other unknown personal reasons that study participants may have had. In the laboratory tests, the working heights were always optimally adjusted to the height of the user, which is only partially possible in the field. Due to their anatomical and physiological dispositions, individuals have different requirements for a standardized or less flexible workplace. Thus, before using exoskeletons, the individual should always be tested and evaluated in relation to the workplace and this should inform the selection of the most suitable exoskeleton model.

### Exoskeleton Characteristic and Use in Everyday Work

We observed a trend towards slim shoulder-supporting exoskeletons, i.e., 11 of the 18 exoskeletons used in the research studies included in this review can be counted to this type of design. Endpoint-based or whole-arm exoskeletons (both *n* = 3) are applicable to a narrower range of tasks due to their complexity and size. We also found that the evaluations carried out were multi-faceted and investigators considered many different objective and subjective parameters. This is mainly due to the heterogeneous requirements that exoskeletons have to meet depending on the individuals who wear them and the tasks they are used to provide support for. In order to achieve an efficient and goal-oriented use, it is important to create a balance between the characteristics of the exoskeleton, the needs of the users and the requirements of the work process (Voilqué et al., 2019). However, based on their experience from a field study, Hensel and Keil (2019) do not recommend using exoskeletons as the sole means of remedying ergonomic constrictions. As exoskeletons fit closely to the body, they are to be considered personal protective equipment according to the German Professional Trade Association for Wood (Berufsgenossenschaft Holz und Metall, 2017) and should therefore be used as a last step in combination with preceding technical and organizational measures such as the use of balancers and workplace rotation. However, as already described, it should be clarified on an individual basis whether the use of an exoskeleton is target-oriented. Due to the small number of field studies currently available and the declining intention to use, it may be more promising to start with a short test phase at suitable workplaces. As mentioned above, these seem to be mainly repetitive tasks (such as on an assembly line) with a high proportion of OHW, static tasks and precision tasks.

### Comparison to Prior Review on Exoskeletons in the Work Environment

Compared to the first review (de Looze et al., 2015) on exoskeletons in the work environment, we noted that the designs of exoskeletons for upper limbs have changed in recent years. With one exception (Naito et al., 2007), only anthropomorphic exoskeletons to support the entire arm (shoulder and elbow joint) or full-body exoskeletons were used. In this review, the included studies mainly focused on shoulder-supporting exoskeletons, some of which were not anthropomorphic. However, of note, some exoskeletons are already available on the market. Similarly, the number of studies and the number of participants is higher in our review as compared to the one published in 2015, and the effectiveness of the exoskeletons that was initially suspected could be confirmed by our review. Furthermore, the focus has also increasingly been placed on passive systems and it was shown that these could achieve similar results as their active counterparts with regard to the relief of muscles. Due to the changed designs and the passive energy supply, less complex and also more cost-effective systems can be used in the work context. In addition, exoskeletons now appear to be more comfortable, which may have positive implications for user acceptance.

### Limitations of the Studies

Overall, it must be noted that studies included in this review differed greatly with regard to study protocols (familiarization period, task, duration, repetitions, etc.) and results are thus comparable only to a limited extend (see also de Bock et al., 2022). A standardized protocol (e.g., Groos et al., 2022b; de Looze et al., 2022) for research examining the use and effectiveness of exoskeletons for OHW would simplify the comparison of the different models for future research studies.

Unfortunately, the majority of participants of the included studies were males. Due to sex differences in anatomy and physiology, it is likely that males and females may differ with regard to the evaluation of exoskeletons. Since exoskeletons by nature have to fit very closely to the body, the accuracy of fit and adaptability, but also the distribution of pressure at the contact points and their position are decisive. Therefore, future studies should pay closer attention to a balanced gender ratio of participants. Furthermore, the conclusions derived from our review mainly apply to individuals aged 40 years and younger, and the average age of participants was over 40 years in only 5 of the included studies. However, since the current average age of workers in Germany increased from 41.9 to 44.1 years between 2004 and 2017, and is predicted to increase to up to 45.2 years by 2030, the studies are not entirely reflective of the working population (German Federal Institute for Population Research, 2019). In addition, with increasing age, the strength capacity continuously decreases; thus, the use of exoskeletons for older workers may particularly be useful–if and when proven effective-in order to maintain work-related performance in old age (Keller and Engelhardt, 2014). The likelihood of developing MSDs also increases with age (March et al., 2014). However, in most of the studies, only healthy individuals were included and, to the best of our knowledge, no study to date explicitly examined the effectiveness of upper-limb exoskeletons in persons with MSDs, albeit the use of exoskeletons in individuals with MSDs may have many benefits in terms of reintegration into the work process or even maintaining the ability to work. Thus, whereas exoskeletons may also have various benefits to young workers (e.g., reduced fatigue) as outlined above, future studies should also include older participants as well as persons with pre-existing medical conditions. Another main limitation pertains to the small number of field studies (i.e., workplace setting/environment), as the majority of studies was conducted in the laboratory setting. For a successful evaluation and introduction of exoskeletons in the field and in order to increase evidence and quality of findings, more research examining the effectiveness of exoskeletons in the workplace setting is critically important. In addition, attention must also be paid to the extent as to which results from the laboratory can be transferred to the field (see also de Bock et al., 2021). Finally, it should be noted that conclusions about potential long-term effects of the use of exoskeletons in the workplace environment can only be drawn to a limited extent based on the current state of research. In fact, we are aware of only three field studies that examined the effectiveness of exoskeletons after a wearing period of more than one working day. Thus, more prospective studies on the potentially preventive effects or changes in movement behavior due to exoskeletons are needed.

### Limitations of the Review

This review has some limitations. First, the search, selection process, and data extraction were performed by only one author (TM), which does not entirely comply with the requirements of the PRISMA Statement. As a result, bias may have been present during the selection and evaluation processes. Second, for the systematic search, the term “exoskeleton” was used as the main keyword. This term has only emerged in the last 10–15 years and is now well established. It is thus possible that previous studies with similar approaches that used other terminologies, such as “personal lift augmentation device (PLAD)", “muscle suit” or “wearable device” may not have been detected. However, the review by de Looze et al. (2015) shows that publications prior to 2015 are generally restricted to preliminary studies with limited power to report effects.

## Conclusion

The number of upper-limb exoskeletons available for use in the working environment has increased significantly over the last few years. Most studies focus mainly on tasks at overhead level when designing and using exoskeletons. Preliminary evidence shows that use of upper-limb exoskeletons may provide relief for the user with regard to their target movement, with effects being stronger in the laboratory than field setting. Furthermore, it is recommended that an individual wearing an exoskeleton should always be assessed and evaluated beforehand in relation to the workplace and the respective exoskeleton. At this point, exoskeletons cannot be considered a tool for mass use, but rather should be used carefully in employees with a confirmed need. The current body of research on the effectiveness of exoskeletons in a workplace setting is limited by short wearing times as well as limited sex and age distribution of participants which are not representative of the majority of workers. Future studies should thus apply longer exoskeleton wearing times and follow-up durations, as well as include more women, older individuals and persons with pre-existing medical conditions.
